# Takotsubo Cardiomyopathy in a Post-COVID Case

**DOI:** 10.7759/cureus.45514

**Published:** 2023-09-18

**Authors:** Amanpreet S Wasir, Ravi Kalra, Puneet Verma

**Affiliations:** 1 Cardiology, Bharati Vidyapeeth University and Medical College, Pune, IND; 2 Cardiology, ACE Heart and Vascular Institute, Mohali, IND

**Keywords:** left ventricle, takotsubo cardiomyopathy, regional wall motion abnormality, diastolic dysfunction, covid-19

## Abstract

Takotsubo cardiomyopathy (TTC) is a unique heart disease that mimics the clinical presentation of acute coronary syndrome and is seen more commonly in post-menopausal females. Here, we report a case that presents an ideal documentation of TTC depicting its characteristic clinical features and possible outcomes. TTC usually culminates in a complete reversal of both systolic and diastolic dysfunctions, however in our case of a post-COVID scenario, the persisting, rather worsening diastolic dysfunction might be a residual manifestation of COVID-19 myocarditis. Recent reports have found an increasing prevalence of TTC amidst the COVID-19 pandemic possibly as a result of the emotional and physical stress, and subsequent catecholamine surge caused by the virus in such patients. There might exist an independent association between TTC and the COVID-19 virus. Increased clinical evidence is required to establish the strength of this relationship, if any.

## Introduction

Takotsubo cardiomyopathy (TTC) is best described clinically as transient left ventricular apical ballooning and dysfunction without the presence of obstructive coronary artery disease [1,2]. TTC has a multi-factorial pathophysiology with the majority of the patients classically presenting with some kind of 'stress' [2,3,4]. Amidst the recent outbreak of the SARS-CoV-2 virus, it is now well-known that the virus affects the cardiovascular system in various ways [2,5]. The cardiovascular manifestations of the COVID-19 virus include myocarditis, microvascular damage, endothelial dysfunction, and structural and functional abnormalities of worsening cardiac dysfunction, among others [2,5]. Recent studies have found an increasing prevalence of TTC as an 'acute coronary syndrome mimicker' in patients affected by the COVID-19 virus [2,6,7]. The occurrence of TTC in patients with COVID-19 is attributed largely to the emotional and physical stressors that are incubated during the COVID-19 outbreak which in turn trigger a cytokine storm and subsequent catecholamine surge in the body responsible for pre-disposing such patients to TTC [2,3,6,7]. However, the etiopathogenesis and clinical spectrum of the relationship between TTC and the COVID-19 virus remains ill-defined and requires more clinical evidence to establish its strength as a potential independent association.

## Case presentation

**Figure 1:**
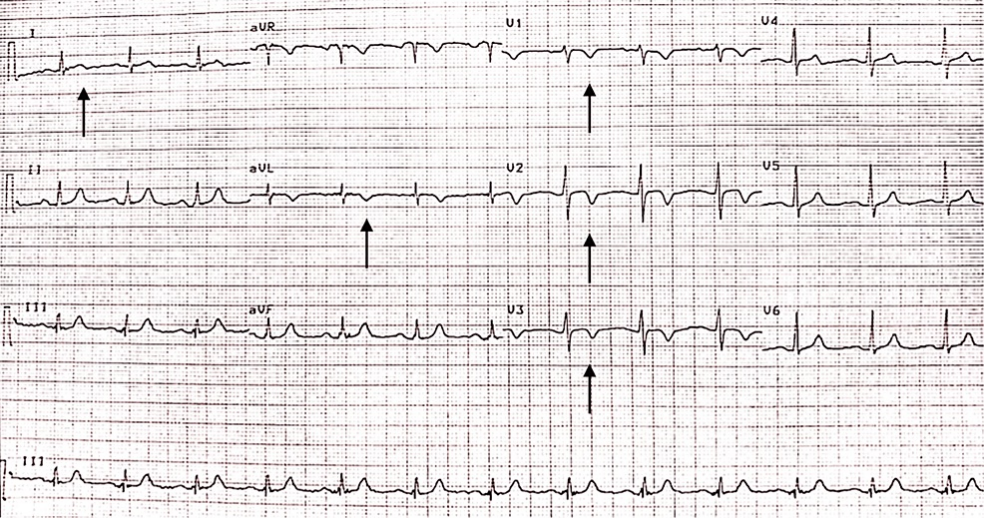
Electrocardiogram showing bi-phasic T wave in lead I and symmetrical T wave inversion in leads V1 to V3 and aVL.

A 68-year-old female presented to the emergency ward of a tertiary care hospital with complaints of sudden onset chest pain, dyspnea, and palpitations. She had a history of hypothyroidism, hypertension, and diabetes, but no significant family history or past history of any cardiovascular disease. She gave a history of mild COVID-19 infection a month ago which was managed symptomatically at home. She recovered over the next two weeks but began to complain of breathlessness on exertion with subsequent relief on taking rest, and bearable exertional chest pain for four to five days prior to admission. Her vitals were: pulse- 69 bpm, blood pressure- 112/56 mmHg, respiratory rate- 20/min, O2 saturation- 95%, afebrile. Electrocardiogram revealed bi-phasic T wave in lead I and symmetrical T wave inversion in leads V1 to V3 and aVL (Figure 1).

**Figure 2:**
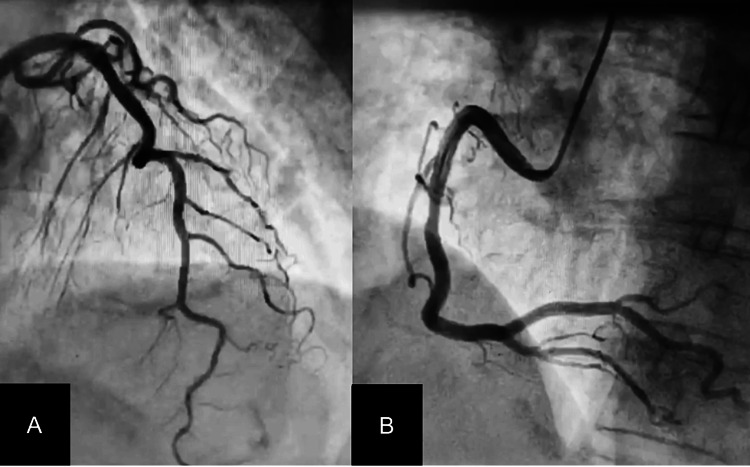
(A) Coronary angiogram showing normal left coronary artery. (B) Coronary angiogram showing normal right coronary artery.

Biochemical tests revealed troponin T level to be 137 ng/L (n= <50 ng/L). Coronary angiography revealed normal epicardial coronary arteries (Figure 2), thereby ruling out coronary artery disease.

**Figure 3:**
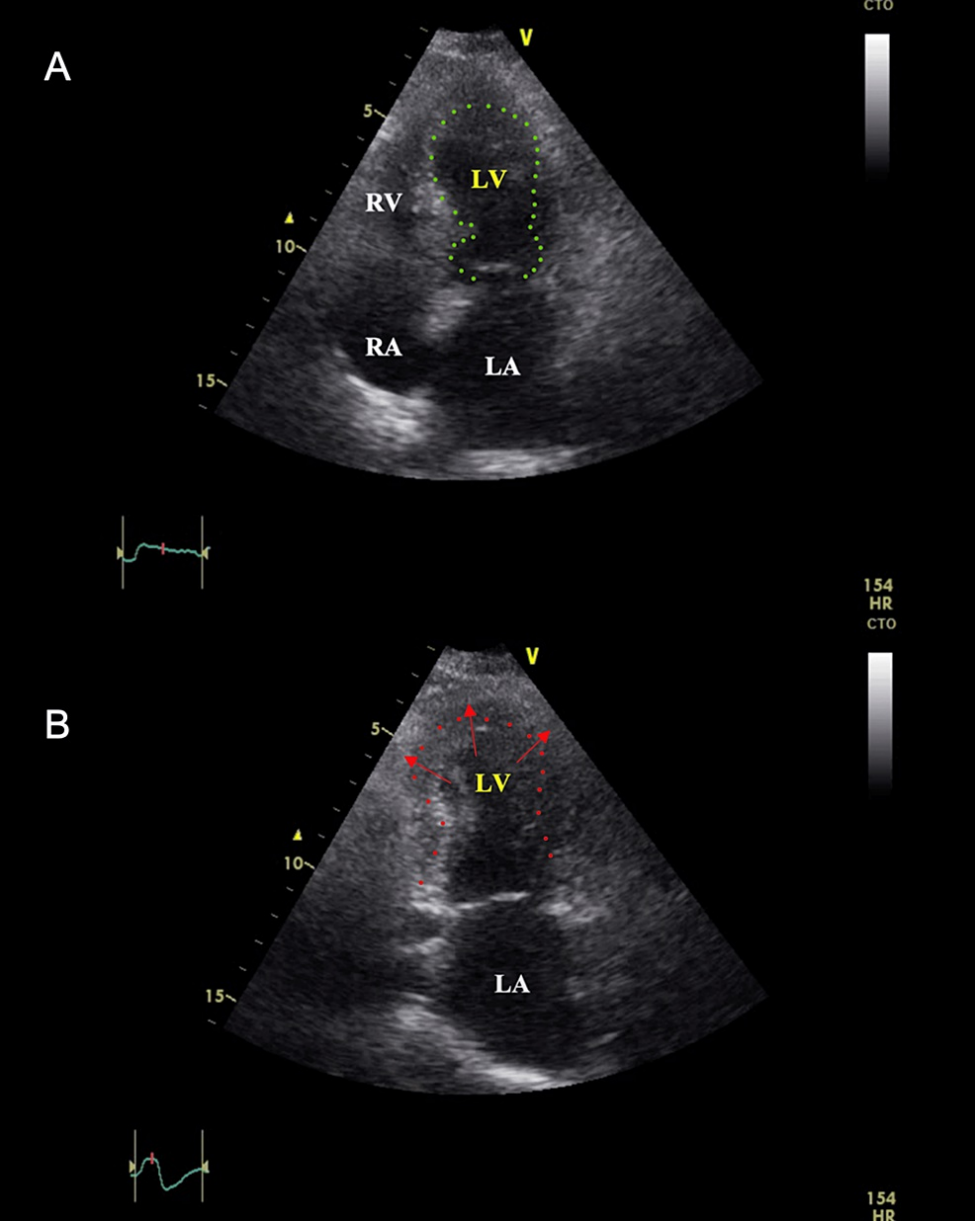
(A) Echocardiogram: apical four-chamber view showing left ventricular apical ballooning. (B) Echocardiogram: apical-two chamber view showing normal left atrium and abnormal expansion of left ventricle. Also noted, left ventricular regional wall motion abnormality-hypokinesia of mid anterior, all apical & mid segments interventricular septum.

An echocardiogram revealed an abnormal appearance of the left ventricle (LV) (Figure 3) and regional wall motion abnormalities at rest with left ventricular ejection fraction= 40%. Clinical diagnosis of TTC was thus made, using the reference of the diagnostic criteria proposed by the Mayo Clinic [3].

**Figure 4:**
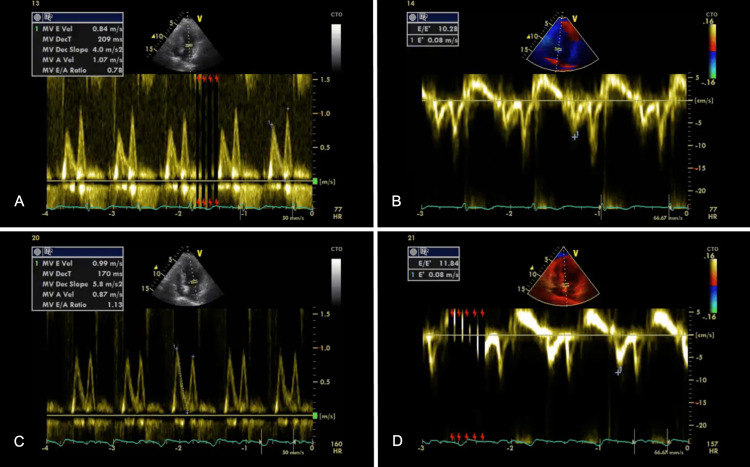
(A and B) - differences in mitral valve and tissue Doppler findings obtained on a subsequent time lapse of four days. On admission, depicting indices; MV E Velocity= 0.84 m/s, MV A Velocity= 1.07 m/s, MV E/A Ratio= 0.78, E/E’ Ratio= 10.28 suggestive of grade I diastolic dysfunction. (C and D) - Pre-Discharge, depicting indices; MV E Velocity= 0.99 m/s, MV A Velocity= 0.87 m/s, MV E/A Ratio= 1.13, E/E’ Ratio= 11.84 suggestive of grade II diastolic dysfunction.

The patient was managed with beta-blockers, angiotensin-converting enzyme (ACE) inhibitors, statins, anti-platelets, and diuretics. The patient improved symptomatically and was kept under observation. After three days, a repeat echocardiogram pre-discharge showed no regional wall motion abnormalities with improved left ventricular ejection fraction= 57% following which the patient was discharged on regular follow-up advice. The mitral valve and tissue Doppler findings suggest worsening of grade I to grade II diastolic dysfunction of the left ventricle with abnormal values of mitral valve diastolic blood flow which have not reversed back to normal values even after a significant time lapse post-admission (Figure 4). At the one-month follow-up, the patient was asymptomatic, involved in regular home activities of daily living, and walking normally with normal effort tolerance.

## Discussion

The overall prevalence of TTC is studied to be 1-2% in cases of suspected acute myocardial infarction [3,8]. It has been found that the majority of patients diagnosed with TTC are above the age of 50 years, [9] with a female-to-male ratio of 9:1, [10] indicating that elderly age and female sex are strong independent risk factors of TTC. COVID-19 infection is known to cause a 'cytokine storm' with an associated catecholamine surge in those moderately to severely affected by the virus, which in turn results in predisposing the occurrence of TTC in such patients [2,6,7]. The prevalence of TTC changed from an initial pre-pandemic range of 1.5-1.8% to 7.8% (n=1914 acute coronary syndrome patients studied across four distinct pre-pandemic timelines) during the pandemic according to a recent retrospective study [7]. Moreover, the impact of the COVID-19 pandemic on physical, psychological, mental, emotional, personal, and social domains has been enormous, which has adversely affected the mental well-being of the community by many-folds, leading to emotional and physical stress, both of which are identified as strong etiological factors for developing TTC [2,11].

In our patient, the left ventricular ejection fraction which had dropped to a low 40% increased to a normal 57% indicating the quick-reversible nature of TTC. Regional wall motion abnormalities of the left ventricle including hypokinesia of mid anterior, apical & mid segments of the interventricular septum also seem to have resolved pre-discharge as were seen previously. The mitral diastolic blood flow has shown a positive trend with a gradually increasing E/A ratio since the episode of TTC from 0.78 to 1.1 pre-discharge. The E/E’ ratio which is indicative of diastolic dysfunction increased from 10.28 initially to 11.84 pre-discharge, suggesting a progressive left ventricular diastolic dysfunction (Figure 4). Importantly even though there is nearly complete reversal of LV systolic dysfunction, grade I diastolic dysfunction has worsened to grade II, suggesting a possible pathological state of the myocardium in TTC, possibly aggravated by the patient's COVID-19 infection, though a baseline derangement of echocardiographic and tissue doppler findings suggesting a persisting diastolic dysfunction in view of age, hypertension and diabetes remains a possibility. In this particular case, there were no other potential triggers found on history and clinical examination that might predispose the development of TTC in our patient. Thus, there exists a possibility of a potential relationship between the previous COVID-19 viral episode and the subsequent development of TTC after approximately a month's duration in our patient. The long-term significance of this still remains unclear, thus the patient needs to be constantly monitored with regular follow-ups.

TTC, well-known for its completely reversible nature, may sometimes lead to persistent diastolic dysfunction, which might have fatal complications in the long run [12]. Herein, even though there may be complete recovery on macroscopic grounds, the microscopic recovery is comparatively much slower and delayed [12]. The systolic and diastolic function may continue to remain abnormal in TTC patients even after the initial ‘recovery phase’ in spite of LV morphology and systolic function being normal [13]. A recent study (n=205; mean age=70±12years; 95% female) showed that the diastolic function improved only in 28% of TTC cases during the recovery phase (mean 38±16 days after admission), whereas, in the remaining 72%, the diastolic function was either unchanged or worsened [14]. Thus, the recovery phase might be delayed or may be incomplete in some TTC cases following its acute onset, and this remains to be studied further.

Lastly, there is a paucity of clinical cases which is required to correctly establish the strength of the association between TTC and the COVID-19 virus. An increased prevalence with fuelled research could prove to be the missing link in defining the severity of this association, which will positively reflect in the early identification of cases and better management strategies aimed at improving patient outcomes.

## Conclusions

We present a patient of takotsubo cardiomyopathy (TTC) in a post-COVID-19 scenario wherein the persistent rather worsening left-ventricular diastolic dysfunction might possibly be a result of the catecholamine surge caused by the virus. Clearly, more evidence is needed to strengthen this association. Recent studies have revealed a higher prevalence of TTC associated with viral contagion, possibly due to different physical and emotional stressors caused during the pandemic. Positive efforts in bridging the gaps between the etiopathogenesis of this relationship will reflect in early diagnosis and targeted treatment strategies aimed at the betterment of patient outcomes.
